# Morphology of Fumes from Hybrid Laser–Arc Welding of X5CrNi18-10 Stainless Steel

**DOI:** 10.3390/ma18245534

**Published:** 2025-12-09

**Authors:** Janusz Adamiec, Joanna Wyciślik-Sośnierz, Jolanta Matusiak, Michał Urbańczyk, Marcin Lemanowicz, Robert Kusiorowski, Anna Gerle

**Affiliations:** 1 Faculty of Materials Engineering and Digitalisation of Industry, Silesian University of Technology, 40-019 Katowice, Poland; janusz.adamiec@polsl.pl; 2 Łukasiewicz Research Network—Upper Silesian Institute of Technology, 44-100 Gliwice, Poland; jolanta.matusiak@git.lukasiewicz.gov.pl (J.M.); michal.urbanczyk@git.lukasiewicz.gov.pl (M.U.); 3 Faculty of Chemistry, Silesian University of Technology, 44-100 Gliwice, Poland; marcin.lemanowicz@polsl.pl; 4 Łukasiewicz Research Network—Institute of Ceramics and Building Materials, 31-983 Cracow, Poland; robert.kusiorowski@icimb.lukasiewicz.gov.pl (R.K.); anna.gerle@icimb.lukasiewicz.gov.pl (A.G.)

**Keywords:** welding fume, morphology, stainless steel, hybrid welding, laser diffraction, SEM-EDS (Scanning Electron Microscopy with Energy Dispersive Spectroscopy)

## Abstract

Stainless steels are widely used across many industrial sectors, including the fabrication of welded structures. The most common methods for joining these materials are arc welding processes. Increasing demands for higher weld quality and process efficiency have led to a growing adoption of laser-based technologies in industry. One of the most frequently applied techniques is hybrid laser–arc welding (HLAW), which combines two heat sources—the laser beam and the electric arc—acting simultaneously. For new, innovative joining technologies, a critical factor in their implementation is their impact on the environment and human health. This article presents the results of a study on the morphology of fumes emitted during the HLAW of X5CrNi18-10 (1.4301) stainless steel. Laser diffraction and scanning electron microscopy were used to characterize the fume morphology. The International Agency for Research on Cancer has classified welding fumes as a carcinogenic agent to humans. The results revealed that more than 20% of particles generated during hybrid welding belong to the most hazardous fraction, as they can penetrate beyond the laryngeal region. These particles exhibit a homogeneous elemental distribution, with the chromium content standing at approximately 20% and nickel nearly 10%.

## 1. Introduction

During welding, dust (a two-phase condensation aerosol, consisting of a mixture of particulate matter—fume—and gases) is emitted into the working environment [1,2]. The particulates are formed through the condensation and oxidation of metal vapors in the arc and weld pool region [3]. Liquid metal appears as very small droplets, with metal vapors as elements and oxides, and also as volatile compounds and elements [4]. The formation mechanisms of welding fumes were described by M. Gonser and T. Hogan [3].

The spatter of fine droplets occurs when a liquid metal droplet strikes the weld pool face, which pushes the droplets out. Upon contact with oxygen, an oxidation reaction takes place. The melting of liquid metal also generates metal vapor, which reacts with oxygen to form metal oxides. In some cases, volatile metal particles evaporate and undergo oxidation upon contact with oxygen. This applies to elements characterized by low evaporation temperatures. Coagulation, in turn, leads to the merging of individual particles into larger agglomerates that form a continuous phase [3].

The International Agency for Research on Cancer (IARC), according to its guidelines, classified welding fumes as a carcinogenic agent to humans [5]. The risk associated with the emission of hazardous substances into the working environment depends not only on the total amount of fumes generated but also on the concentration of specific components within the fumes [6,7]. It should also be noted that the toxicity of welding fumes is determined by the chemical form in which an element occurs and its valence [8,9].

Another factor affecting the toxicity of welding fumes is the structure and size of the particles, as particle size determines the depth of their penetration into the human body [10,11,12]. The respirable and tracheal fractions are particularly hazardous to human health; these fractions include particles with dimensions smaller than 10 µm [13,14]. Thus, the size of fume particles is a key factor in determining their interaction with lung epithelial cells and, consequently, their deposition efficiency in different regions of the lungs, as described in [15,16,17,18]. This explains the ongoing interest of researchers worldwide in the structure and dimensions of fume particles.

Researchers from the Far Eastern Federal University in Vladivostok reported the results of a study on welding fume particles generated during the manual metal arc (MMA) welding of structural and stainless steels. Morphological analysis revealed the presence of fume particles in several forms: spherical particles, nuclear–shell structures, perforated particles, sharp-edged particles, and tree-shaped (coral) agglomerates. Their average diameter was 5 nm, placing them in the category of nanoparticles hazardous to human health [19].

A study conducted by T. Brand, K. Lenz, U. Reisgen, and T. Kraus from the University of Aachen showed that during the shielded metal arc welding (MMA) of austenitic steel 1.4301, 24% of the fume particles were smaller than 100 nm and 10% were smaller than 50 nm. In contrast, during metal active gas (MAG) welding, 54% of the fume particles were smaller than 100 nm and 16% were smaller than 50 nm [20].

Researchers from the National Institute for Occupational Safety and Health in Morgantown, USA, focused on an assessment of fume particle size both in the welder’s breathing zone (30 cm away from the welding arc) and in the surrounding area encompassing adjacent workstations (200 cm away from the welding arc) [21]. This was analyzed during the MAG welding process of unalloyed A36 steel. The welding fume samples presented by Cena, Chen, and Keane showed that, at a distance of 30 cm from the welding arc, particles were primarily spherical (0.5–4.0 μm in diameter) and included fine agglomerates. The study also revealed that the concentration of welding fumes at 30 cm from the arc was five times higher than that measured at 200 cm [21].

In turn, a research team supervised by J. M. Antonini determined the fumes generated during A36 steel MAG welding using solid wires of ER70S-6 (carbon steel) and E308LSi (stainless steel) grades [22]. Fume particles consisted, mostly, of fine grains with equivalent diameters ranging from 0.1 to 1.0 μm. The aerodynamic diameter corresponding to the median mass distribution was calculated to be 0.3 μm for fumes generated with carbon steel wire and 0.25 μm for fumes generated with stainless steel wire.

Chemical analysis performed using inductively coupled plasma atomic emission spectroscopy (ICP-AES) on fumes generated during welding with ER70S-6 wire revealed the presence of mainly iron and manganese. In turn, fumes from welding with E308LSi wire contained chromium and nickel in addition to iron and manganese (Table 1). Thus, compounds with proven carcinogenic effects were present in the fumes generated during stainless steel welding [23,24,25].

Mei et al. from the Royal Institute of Technology in Stockholm performed a particle size analysis of welding fumes, dividing them into nano- (10–170 nm) and micro-scale (0.6–2.5 µm) fractions, and determined the chemical composition of fumes during MAG welding with solid and flux-cored wires of austenitic stainless steel X2CrNi18-9 (1.4307) and duplex steel X2CrMnNiN21-5-1 (1.4162) [26]. The study revealed that, despite the division into fractions directly in the tested air, each fraction was composed mainly of nano-scale particles that subsequently formed clusters. It was shown that most of the particles exhibited a nearly spherical shape, although some irregularly shaped particles were also observed. The smallest fume particles identified were approximately 6 nm in diameter. Chemical composition analysis confirmed the presence of iron, manganese, silicon, chromium, and nickel in the fumes, all occurring in oxide form.

The authors of publication [27] analyzed the morphology of fumes generated during the MAG welding of corrosion-resistant steels X2CrNi18-9 (1.4307) and X2CrMnNiN21-5-1 (1.4162) using solid and flux-cored wires. Their study demonstrated the presence of welding fume particles with dimensions ranging from 20 to 30 nm [27].

The study involved analyzing the content of iron, chromium, manganese, and nickel in the welding fume. Table 2 presents the chemical composition of fumes for selected material combinations, determined using atomic absorption spectroscopy (AAS).

During MAG welding with solid wire, the content of these elements exceeded 46% and was 2.5 times higher than during welding with flux-cored wire. This difference is attributed to the presence of elements in flux-cored wires, such as fluorine, sodium, potassium, and titanium. XPS (electron spectroscopy) analysis showed that the metals listed in Table 2 were present in oxide form [27], confirming the findings reported in publication [7] as well as the authors’ own research.

An analysis of the chemical composition of welding fumes in numerous studies has highlighted the hazards associated with welding corrosion-resistant steels—specifically, the emission of chromium compounds, particularly chromium(VI) and nickel, which have established carcinogenic effects in humans [28,29].

An analysis of the current state of the issue has shown that the characteristics of welding fumes are predominantly associated with arc welding methods, which remain the most commonly used in industry [30,31,32,33,34,35,36,37]. However, statistics indicate that an increasing number of companies are adopting laser welding, which, despite requiring high financial investment, allows for the precise joining of components and the obtainment of repeatable joints characterized by low deformation and a narrow heat-affected zone [38,39]. In addition to laser welding, another process that employs a laser beam is hybrid laser–arc welding (HLAW), a novel joining technique. This innovative method, with considerable application potential, can be used for welding both unalloyed and corrosion-resistant steels. The development and implementation of modern welding technologies respond to the increasing industrial demands for high-quality welded joints and efficient welding processes. However, these advances should be accompanied by an assessment of the environmental aspects of the process. To date, no data are available on the morphology of fumes generated during hybrid laser–arc welding. To the best of the authors’ knowledge, this publication and the research presented in [40,41] represent the first studies to focus specifically on this aspect. Understanding the structure and particle size of welding fumes is crucial due to its proven carcinogenic effects in humans [5,42]. Welders face particular health risks when working with austenitic steels, as their primary alloying elements are chromium and nickel. Compounds of these elements present in welding fumes are classified among substances with proven or probable carcinogenic effects [7,42]. To date, no studies have examined the morphology, shape, and size of fume particles generated during the hybrid laser–arc welding of corrosion-resistant steels of the 1.4301 grade; this paper presents such a detailed analysis.

## 2. Materials and Methods

The analysis of fume morphology was conducted on samples from the hybrid welding of stainless steel, grade X5CrNi18-10 (1.4301) [43]. The chemical composition and mechanical properties of this steel grade are presented in Table 3 and Table 4.

Grade 1.4301 (X5CrNi18-10) is an austenitic stainless steel characterized by good weldability, corrosion resistance, and ductility. It is resistant to most chemical compounds [44]. The widespread application of this steel grade across multiple industrial sectors necessitates its joining. Welding remains the dominant method for joining corrosion-resistant steels. The most commonly used welding techniques for these steels include gas metal arc welding with inert or active shielding gas (MIG/MAG); however, laser and hybrid welding (HLAW) methods are becoming increasingly popular. This paper focuses on the analysis of fumes generated during hybrid welding.

Hybrid laser–arc welding (HLAW–laser + electric arc) combines two heat sources—a laser beam and a conventional arc—in a single process [45]. During hybrid welding, the upper edges of the sheets are melted by the heat generated by the arc, whereas the laser beam enables deeper material penetration and melting [45]. Hybrid welding can be performed in two configurations: A–L and L–A. In the A–L (arc-leading) configuration, the electric arc precedes the laser beam, whereas in the L–A (laser-leading) configuration, the arc follows the laser beam.

As the filler metal during the hybrid welding of steel 1.4301, a solid wire of 308L-Si grade with a diameter of 1.2 mm (G 19 9 L Si according to EN ISO 14343 [46]) was applied. Argon was used as the shielding gas (I1 according to EN ISO 14175 [47]). Morphology analysis was performed on fumes generated with a laser beam power of 6500 W and a welding speed of 1.5 m/min, using a welding current of 250 A and an arc voltage of 28 V. The process was carried out in an A–L configuration. The application of these technological parameters enables the production of joints meeting quality level B according to EN ISO 13919-1 [48].

**Figure 1:**
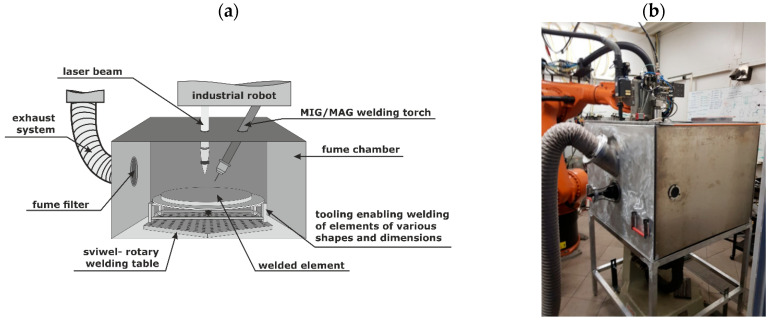
Welding station for fume emission rate determination during hybrid welding: (**a**) scheme of workstation, (**b**) workstation designed in the Centre of Welding.

The gravimetric method constitutes the basis for determining fume emission rates and their subsequent characterization. The principle is to collect the fumes on filters during welding in a purpose-built research station at the Centre of Welding of the Upper-Silesian Institute of Technology (Figure 1).

The main element of the research station was a chamber, inside which the welding process took place. The chamber was made of 5754 aluminum alloy with a thickness of 4 mm. Its dimensions are as follows: length and width of 920 mm, and a height of 760 mm, resulting in a chamber volume of 0.64 m^3^. The chamber design prevents the escape of contaminants to the external environment. A suction port, in which the filter is placed, is located on the side of the chamber. The chamber remains fixed while the welded specimen, mounted on a rotary table, rotates (Figure 1). PTM-B filters with a diameter of 150 mm were used in the study [49].

A methodology for evaluating the hazard of welding fumes, including their morphology, was developed by the authors. The structure of the welding fumes (shape and dimensions) was analyzed using scanning electron microscopy (SEM) and laser diffraction. During welding in the specially designed chamber, fumes generated by the process were extracted and collected on a measurement filter, and subsequently transferred for analysis.

A Mira 3 electron microscope (Tescan, Brno, Czech Republic), equipped with an energy-dispersive spectrometer (EDS) system and AZtec Automated software v.3.1, was used for welding fume microstructure analysis. The accelerating voltage was set to 15 kV in either backscattered electron (BSE) or secondary electron (SE) mode for image acquisition. The electron beam diameter was 11 nm. Fume samples were deposited on conductive carbon tape and additionally coated with a conductive layer of graphite using a Quorum Q150R (Quorum Technologies Ltd., Lewes, UK) ES device.

To determine the particle size distribution, a laser diffraction technique is employed, utilizing the phenomenon of light scattering by particles. Particle size distribution is assessed by measuring the angular variation in the intensity of light scattered as a laser beam passes through the sample; the smaller the particle, the greater the scattering angle [50]. The recorded spectrum of scattered light is analyzed to obtain the particle size distribution. The studies were conducted using an Analysette 22 laser particle analyzer (Fritsch GmbH—Milling and Sizing, Weimar, Germany), which allows the measurement of particle sizes in the range of 0.16–2000 µm.

## 3. Results

### 3.1. Welding Fume Particles’ Microstructure

**Figure 2:**
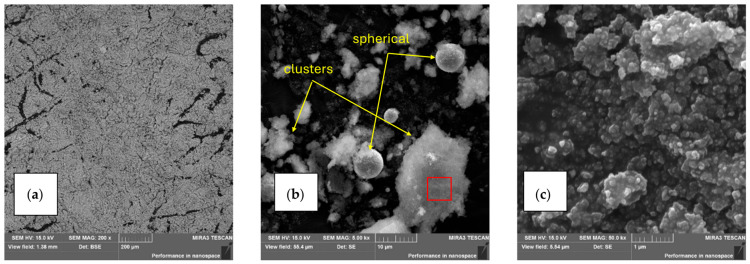
Fume particles generated during hybrid welding of steel 1.4301. (**a**) Fine-grained particles; (**b**) individual particles (irregular and near-spherical shapes) and larger clusters (agglomerates)—arrows indicate fume particles; the boxed region in (**b**) corresponds to the higher-magnification image shown in (**c**); (**c**) clusters with distinctly visible particles.

Figure 2 shows fume particles generated during the welding of steel 1.4301, at magnifications ranging from 200× (a) to 50,000× (c). The fumes appear either as individual particles with elongated or near-spherical shapes, or as chains and agglomerates (Figure 2).

**Figure 3:**
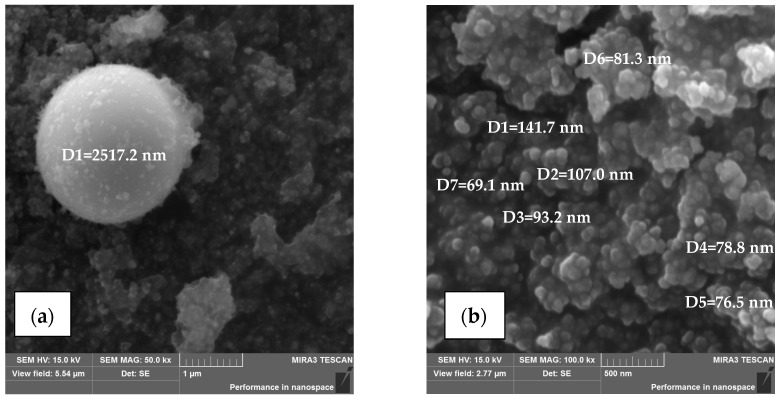
Dimensions of fume particles generated during hybrid welding of steel 1.4301: (**a**) particle with a spherical shape with a diameter of 2.5 µm, (**b**) fine spherical particles with diameters of 70–110 nm.

Particle size measurements conducted during SEM observations (Figure 3) revealed the presence of spherical fume particles with a diameter of approximately 2.5 µm (Figure 3a). At higher magnifications, fume particles with sizes in the range of 70–110 nm were observed (Figure 3b).

**Figure 4:**
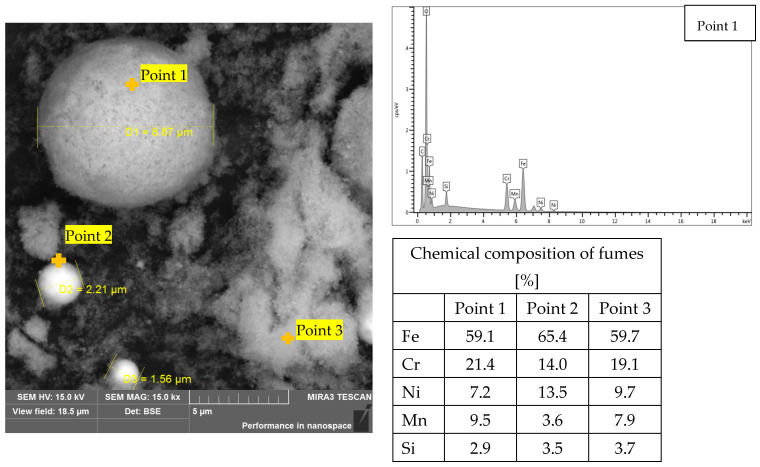
Chemical composition and morphology analysis of fumes generated during hybrid welding of austenitic steel 1.4301.

For selected fume samples from the hybrid welding of 1.4301-grade steel, chemical composition analysis was performed using EDS in combination with particle size analysis. Representative results are shown in Figure 4.

Figure 4 shows spherical and irregularly shaped fume particles, as well as their larger clusters. These agglomerates are formed due to the increased mobility of particles at high temperatures. Chemical composition analysis indicated the presence of iron, chromium, nickel, manganese, and silicon in the fumes, consistent with the results reported in previous publications [22,26]. It was found that the main component of the fumes from the hybrid welding of steel 1.4301 is iron (approximately 60%). The chemical composition of particles at points 1 and 3 was observed to be similar. These particles consisted of nearly 60% iron, 20% chromium, 8% nickel and manganese, and approximately 3% silicon. The analyzed area also contained particles belonging to the respirable fraction, with sizes ranging from 1.5 to 2.2 µm. For example, a spherical particle with a diameter of 2.2 µm (Figure 4, measurement point 2) consisted of more than 65% iron and nearly equal amounts of chromium and nickel (approximately 14% each). The contents of manganese and silicon in this particle were equal, standing at 3.5%.

**Figure 5:**
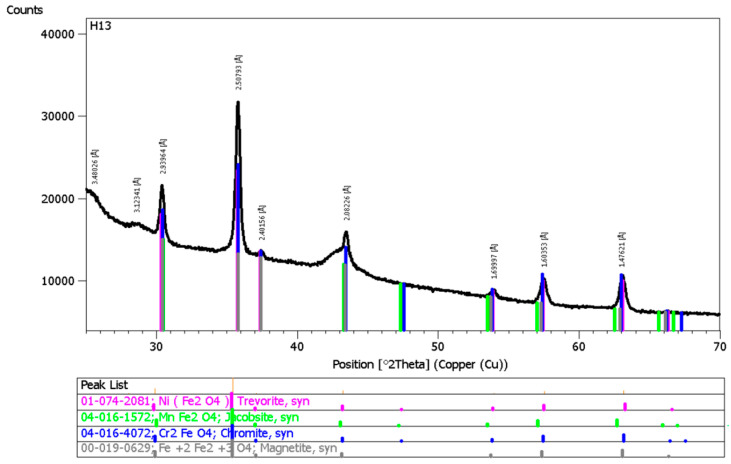
X-ray diffractogram of fumes generated during hybrid welding of steel 1.4301.

Chemical composition analysis at all measurement points revealed the presence of oxygen (Figure 4), indicating that the detected elements were present as oxides. This was verified by phase identification using X-ray diffraction (XRD) (Figure 5), which demonstrated the presence of specifically spinels with the general formula AB_2_O_4_, where Cr, Mn, Ni, and Fe occupy the A and B sites [7,40].

The following phases were identified: chromium(III) iron(II) oxide (Cr_2_FeO_4_—Cr_2_O_3_/FeO), iron(III) manganese(II) oxide (MnFe_2_O_4_—MnO/Fe_2_O_3_), nickel(II) iron(III) oxide (NiFe_2_O_4_—NiO/Fe_2_O_3_), and iron(II,III) oxide (Fe_3_O_4_—FeO/Fe_2_O_3_). The main phase present in the fumes was chromium(III) iron(II) oxide (Cr_2_FeO_4_—Cr_2_O_3_/FeO), a compound belonging to the chromium spinel group. These compounds also contain chromium in the hexavalent (VI) oxidation state [51,52]. Chromium(VI) is classified by the International Agency for Research on Cancer (IARC) as a Group 1 substance, i.e., an agent carcinogenic to humans [7].

**Figure 6:**
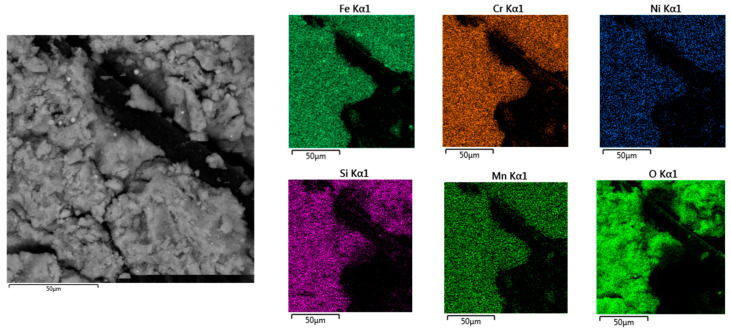
Chemical elements distribution map of fumes generated during hybrid welding of steel 1.4301.

Figure 6 presents a map showing the distribution of chemical elements in a fume sample obtained from the hybrid welding of steel 1.4301.

The fume sample contained both individual particles and larger clusters in the form of chains or agglomerates. The distribution of elements within the analyzed area was homogeneous. Oxygen was also detected on the surface of the fume particles, confirming that the previously identified elements were present in oxide form [41].

### 3.2. Fume Particle Size Assessment

**Figure 7:**
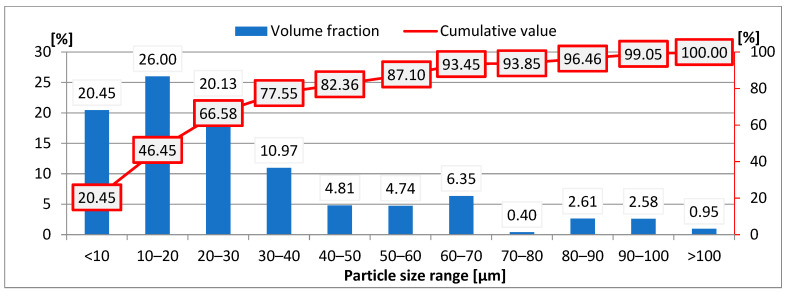
Particle size distribution of fumes generated during hybrid welding of steel 1.4301.

Figure 7 presents a histogram of the particle size distribution for fumes generated during hybrid welding. The most numerous group consisted of particles in the 10–20 μm range, representing a volume fraction of 26% (Figure 7). Another 20.45% of the total volume corresponded to the finest particles—those smaller than 10 μm. The volume fraction of particles in the 20–30 μm range accounted for approximately 20% of the total fumes (Figure 7).

The results indicated that particles smaller than 20 μm constituted 47% of the fume sample, whereas more than two-thirds of the analyzed fumes consisted of particles smaller than 30 μm. In fumes generated during hybrid welding, over 99% of particles were smaller than 100 μm.

To estimate the occupational risk associated with fume exposure, the distribution of the finest fume fraction (particles smaller than 10 μm) was determined. The specification presented in Table 5 includes fume particles belonging to the respirable (<3 μm) and tracheal (3–10 μm) fractions.

An analysis of the results revealed that 20.5% of fumes generated during the hybrid welding of steel 1.4301 penetrate beyond the larynx (tracheal fraction), of which nearly 6.5% are particles capable of reaching and depositing in the ciliated respiratory tract (respirable fraction).

The quantitative distribution of particles within the designated size ranges was determined and is presented in Table 6. It was revealed that in fumes generated during the hybrid welding of stainless steel, nearly 100% (99.97%) of particles were smaller than 10 µm.

In fumes generated during the hybrid welding of steel 1.4301, 99.5% of the particles belonged to the respirable fraction and therefore pose the greatest threat to human health, which is particularly important when taking into consideration the widespread application of 1.4301 stainless steel in industrial sectors including the food, chemical, petrochemical, and construction industries [44]. The results of this study are consistent with data from the literature indicating that only 10–30% of fume particles (depending on the welding process) have a diameter larger than 1 µm. This indicates that more than 70% of welding fume particles belong to the fraction most hazardous to human health [6,53]. Fume particles smaller than 3 µm can penetrate the alveoli of the lungs and enter the bloodstream, contributing to the development of various diseases affecting the lungs, heart, and nervous system [6].

It was shown that fumes generated during hybrid welding, similar to those from arc welding, consisted of individual, near-spherical particles forming larger clusters [21,30]. The chemical composition of fumes from the hybrid welding of steel 1.4301 was comparable to that of fumes from the MAG arc welding of steel A36, consisting of approximately 60% Fe, 20% Cr, and 8.5% Ni, whereas fumes from arc welding contained a higher manganese content—approximately 14% [22]. The comparison of the results indicates that the chemical composition of fumes from the arc and hybrid welding of steel 1.4301 was similar [31]. A difference was observed in the particle size distribution: fumes from hybrid welding contained a higher volume fraction of the finest particles (smaller than 10 µm)—20.45% for hybrid welding compared with 15.70% for arc welding [30].

The results of this study indicate that the technological parameters of hybrid welding, such as wire feed rate, laser beam power, and welding speed, affect the fume emission rate [52]. Adjusting these parameters can reduce the amount of fumes entering the human body, thereby lowering the risk of occupational diseases associated with fume exposure. A detailed discussion of technological parameters’ impact on fume emission rate was provided in [54].

## 4. Conclusions

Based on the analysis of the research results, the following conclusions were drawn:

1. The above-presented study developed a methodology for analyzing welding fume morphology. The methodology includes welding tests conducted in a specially designed research station equipped with a fume chamber to collect fumes on measurement filters. Scanning electron microscopy with energy-dispersive spectroscopy (SEM-EDS) and laser diffraction (LD) were employed to evaluate fume morphology, including particle size, shape, and structure.
2. Fumes emitted during the hybrid welding of 1.4301 stainless steel contained iron, chromium, nickel, manganese, and silicon. The presence of oxygen was also confirmed. X-ray diffraction (XRD) analysis revealed the following spinel phases: Cr_2_FeO_4_, MnFe_2_O_4_, NiFe_2_O_4_, and Fe_3_O_4_.
3. Fume particles generated during the hybrid welding of stainless steel ranged in size from less than 1 µm to 100 µm. Over 20% of the particles belong to the most hazardous fraction due to their morphology. These particles fall within the respirable and tracheal fractions and exhibit a uniform elemental distribution. The fumes contained approximately 20% chromium and nearly 10% nickel.
4. The volume fraction of fume particles smaller than 3 µm, corresponding to the respirable fraction, was 6.5%. In turn, the quantitative share of particles in this fraction was 99.5%.
5. The information obtained on fume morphology, in conjunction with welding technological parameters, can help raise awareness among engineers and technologists regarding health and safety aspects for welders and personnel working near welding stations. The welding of corrosion-resistant steels generates fumes, which poses a significant threat to the environment as well as to the health and safety of workers. The main alloying elements of these steels, namely chromium and nickel, form compounds classified as substances with proven or probable carcinogenic effects.

## Figures and Tables

**Table 1:** Chemical composition of welding fumes [22].

Element	ER70S-6 Solid Wire Grade	E308LSi Solid Wire Grade
	Content [%]	
Fe	82.8	57.2
Mn	15.2	13.8
Cr	-	20.3
Ni	-	8.51
Cu	1.84	0.156
Al	0.165	0.085

**Table 2:** Iron, chromium, nickel, and manganese content in welding fumes generated during MAG welding of corrosion-resistant steels [27].

Designation	Cr [%]	Ni [%]	Mn [%]	Fe [%]	Cr + Ni + Mn + Fe [%]
S1	7.9 ± 0.07	3.5 ± 0.02	11.0 ± 0.09	24.0 ± 0.04	46.4 ± 0.15
F1	2.5 ± 0.07	0.64 ± 0.02	6.9 ± 0.34	7.7 ± 0.41	18.8 ± 0.83
F3	3.6 ± 0.06	0.57 ± 0.02	6.3 ± 0.10	6.7 ± 0.08	17.2 ± 0.25

where (S1) MAG welding of 1.4307-grade steel using solid wire and shielding gas: Ar + 2% CO_2_; (F1) MAG welding of 1.4307-grade steel using flux-cored wire and shielding gas: Ar + 18% CO_2_ + 0.03% NO; (F3) MAG welding of 1.4162-grade steel using flux-cored wire and shielding gas: Ar+18% CO_2_ + 0.03% NO.

**Table 3:** Chemical composition of X5CrNi18-10 (1.4301)-grade steel [43].

Steel Grade	Chemical Composition [%]									
X5CrNi18-10 (1.4301)	**C**	**Si**	**Mn**	**P_max_**	**S**	**N**	**Cr**	**Mo**	**Ni**	**Ti**
	≤0.07	≤1.0	≤2.0	0.045	≤0.015	0.11	17.5–19.5	-	8.0–10.5	-

**Table 4:** Mechanical properties of X5CrNi18-10 (1.4301)-grade steel [43].

Steel Grade	Mechanical Properties		
	Proof Strength R_e0,2_ [MPa]; min.	Tensile Strength R_m_ [MPa]	Elongation After Fracture A [%]
X5CrNi18-10 (1.4301)	210	520–720	45

**Table 5:** Distribution of the volume fraction of the finest fume particles (smaller than 10 μm) generated during hybrid welding of steel 1.4301, including respirable (<3 μm) and tracheal (3–10 μm) fractions.

Fraction	Particle Size [µm]	Fumes from 1.4301 Steel Hybrid Welding	
		Volume Fraction [%]	Cumulative Value [%]
respirable	<1	1.18	1.18
	1–2	2.54	3.72
	2–3	2.63	6.35
tracheal	3–4	2.20	8.55
	4–5	2.56	11.11
	5–6	1.46	12.57
	6–7	1.61	14.18
	7–8	1.82	16.00
	8–9	2.07	18.07
	9–10	2.37	20.45

**Table 6:** Distribution of the quantitative fraction of the finest fume particles (smaller than 10 μm) generated during hybrid welding of steel 1.4301, including respirable (<3 μm) and tracheal (3–10 μm) fractions.

Fraction	Particle Size [µm]	Fumes from 1.4301 Steel Hybrid Welding	
		Quantitative Fraction [%]	Cumulative Value [%]
respirable	<1	94.20	94.20
	1–2	4.45	98.65
	2–3	0.85	99.50
tracheal	3–4	0.24	99.74
	4–5	0.12	99.86
	5–6	0.04	99.90
	6–7	0.03	99.93
	7–8	0.02	99.95
	8–9	0.01	99.96
	9–10	0.01	99.97

## Data Availability

The original contributions presented in this study are included in the article. Further inquiries can be directed to the corresponding author.
